# No more shoulders: Technical modification of Byars' flaps

**DOI:** 10.4103/0970-1591.40629

**Published:** 2008

**Authors:** Eric A. Kurzrock, Nicholas Hellenthal

**Affiliations:** Department of Urology, University of California Davis Children's Hospital, Sacramento, CA, USA

**Keywords:** Hypospadias, plastic, surgery, surgical flaps

## Abstract

The repair of midshaft or more proximal hypospadias generally leads to a deficiency of ventral penile skin. Transposition of dorsal/lateral skin flaps may lead to redundant skin folds or shoulders. In most cases, redundant skin can be avoided by appropriate tailoring and positioning of the flaps. We describe a simple method to correct these folds when they are unavoidable.

## INTRODUCTION

There are three major components to hypospadias surgery: repair of chordee, urethroplasty and remodeling of the preputial, and penile skin. The repair of midshaft or more proximal hypospadias generally leads to a deficiency of ventral penile skin. The most popular techniques for covering the ventral penis entail sagital or transverse incision of the dorsal preputium. The dorsal and lateral preputial/penile skin is then transposed as a bi-pedicle visor flap, as described by Ombredanne and popularized by Nesbit, Byars’ flaps or as a button-hole transposition.[1–4] All these techniques may lead to redundant skin folds (bumps, dog ears, or shoulders) on the lateral aspects of the penis.[5]

These skin folds occur from either bunching of the skin and/or transposition of redundant dorsal preputial skin. In most cases the redundant skin can be diminished with careful placement and tailoring of the flaps. However, in a minority of cases the redundancy of skin seems unavoidable. Simple excision of the folds is dangerous as the underlying blood vessels supply the flaps. To avoid lateral folds, Koltuksuz *et al.* proposed covering the dorsal penis with preputium and transposing the dorsal/lateral penile skin ventrally.[6] This has not become popular possibly due to the poor esthetics of the entire dorsal penis being covered with preputial skin. Kaplan has successfully utilized a preputial island flap for ventral skin coverage which avoids folds.[7]

We have been using a simple technique for correction of redundant skin folds following Byars' flaps. The technique is described and the long-term results are presented.

## TECHNIQUE

After completion of the corporoplasty and urethroplasty, the deficiency of ventral skin is measured. We utilize Firlit collars in all patients.[8] The dorsal preputial/penile skin is divided sagitally for the creation of Byars’ flaps. Excess preputial skin is excised and the flaps are sewn to the coronal margin and to the inferior edges of the Firlit collars. Depending upon the ventral defect, the flaps are sewn to each other in the midline or crossed. In most cases, redundant lateral skin folds are avoided by appropriate tailoring and positioning of the flaps.

**Figure 1:**
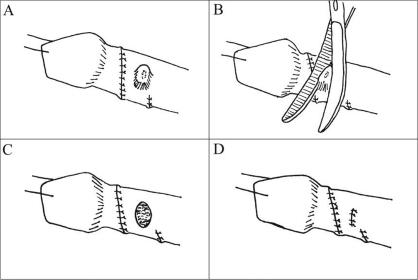
Lateral skin fold (shoulder) from Byars’ flap is demarcated (a) and carefully de-epithelialized (b) without disturbing underlying fat and vasculature. Resulting skin edges (c) are reapproximated with interrupted suture (d)

For those boys left with lateral folds, the overlying skin is marked with a blue pen. The skin folds are de-epithelialized using curved iris scissors with the tips up [Figure 1]. After all the marked epithelium has been excised, the skin edges are reapproximated with interrupted sutures. More than 10 patients have undergone this modification of Byars’ flaps with excellent postoperative penile form one year after surgery. No child has suffered evident ischemia, necrosis, or contraction of ventral skin.

## DISCUSSION

A successful hypospadias repair requires construction of a functional urethra, straightening of the penis, and appropriate skin coverage. There are few options to cover the ventral penis, all of which require transposition of dorsal/lateral skin. The redundant lateral skin folds that occur during skin coverage in some cases can compromise the cosmetic result of the repair. While there have been numerous techniques described and implemented for hypospadias repair, there is little written on the management of redundant lateral skin.

When we first contemplated the problem of redundant lateral skin folds, we thought the underlying fat was the culprit. In some boys who did not require Byar's flaps, we removed the fat (prior to excising the skin flaps). We were surprised to find that most of the fold remained. The excess wrinkled skin turned out to be the main factor creating the folds, not the underlying fat. Our described technique removes this skin. In addition, the reapproximation of the skin edges appears to invert the underlying fat and vasculature inward.

## CONCLUSIONS

Redundant skin folds or shoulders from Byars’ flaps can be avoided in most cases by careful positioning and tailoring of skin flaps. When necessary, de-epithelialization of the redundant skin and reapproximation provides a natural, lasting cosmetic result without compromising the underlying blood supply to the ventral skin.
